# Targeting MicroRNA Function in Respiratory Diseases: Mini-Review

**DOI:** 10.3389/fphys.2016.00021

**Published:** 2016-02-04

**Authors:** Steven Maltby, Maximilian Plank, Hock L. Tay, Adam Collison, Paul S. Foster

**Affiliations:** ^1^Priority Research Centre for Asthma and Respiratory Diseases, Hunter Medical Research Institute, University of NewcastleCallaghan, NSW, Australia; ^2^Department of Microbiology and Immunology, School of Biomedical Sciences and Pharmacy, University of NewcastleCallaghan, NSW, Australia; ^3^Experimental and Translational Respiratory Medicine, Faculty of Health, School of Medicine and Public Health, University of NewcastleCallaghan, NSW, Australia

**Keywords:** microRNA, respiratory diseases, non-coding RNA, antagomir, mimic

## Abstract

MicroRNAs (miRNAs) are small non-coding RNA molecules that modulate expression of the majority of genes by inhibiting protein translation. Growing literature has identified functional roles for miRNAs across a broad range of biological processes. As such, miRNAs are recognized as potential disease biomarkers and novel targets for therapies. While several miRNA-targeted therapies are currently in clinical trials (e.g., for the treatment of hepatitis C virus infection and cancer), no therapies have targeted miRNAs in respiratory diseases in the clinic. In this mini-review, we review the current knowledge on miRNA expression and function in respiratory diseases, intervention strategies to target miRNA function, and considerations specific to respiratory diseases. Altered miRNA expression profiles have been reported in a number of respiratory diseases, including asthma, chronic obstructive pulmonary disease, cystic fibrosis, and idiopathic pulmonary fibrosis. These include alterations in isolated lung tissue, as well as sputum, bronchoalveolar lavage fluids and peripheral blood or serum. The observed alterations in easily accessible body fluids (e.g., serum) have been proposed as new biomarkers that may inform disease diagnosis and patient management. In a subset of studies, miRNA-targeted interventions also improved disease outcomes, indicating functional roles for altered miRNA expression in disease pathogenesis. In fact, direct administration of miRNA-targeting molecules to the lung has yielded promising results in a number of animal models. The ability to directly administer compounds to the lung holds considerable promise and may limit potential off-target effects and side effects caused by the systemic administration required to treat other diseases.

## Introduction

MicroRNAs (miRNAs) are small non-coding RNA molecules that inhibit protein translation from target mRNAs. More than 1000 unique miRNAs are present in the human genome (Lewis et al., 2005; Berezikov, 2011) and miRNAs are thought to modulate expression of >60% of genes (Friedman et al., 2009). MiRNAs function across a wide variety of biological processes and are increasingly recognized as biomarkers for disease diagnosis and potential therapeutic targets for treatment. In respiratory diseases, there is emerging evidence that altered miRNA expression modulates disease processes and ultimately disease pathogenesis. In this mini-review, we will briefly summarize the current understanding of how miRNAs are processed and function, their roles in respiratory diseases and techniques available to modulate miRNA function *in vivo*.

## MiRNA processing and function

**Figure 1:**
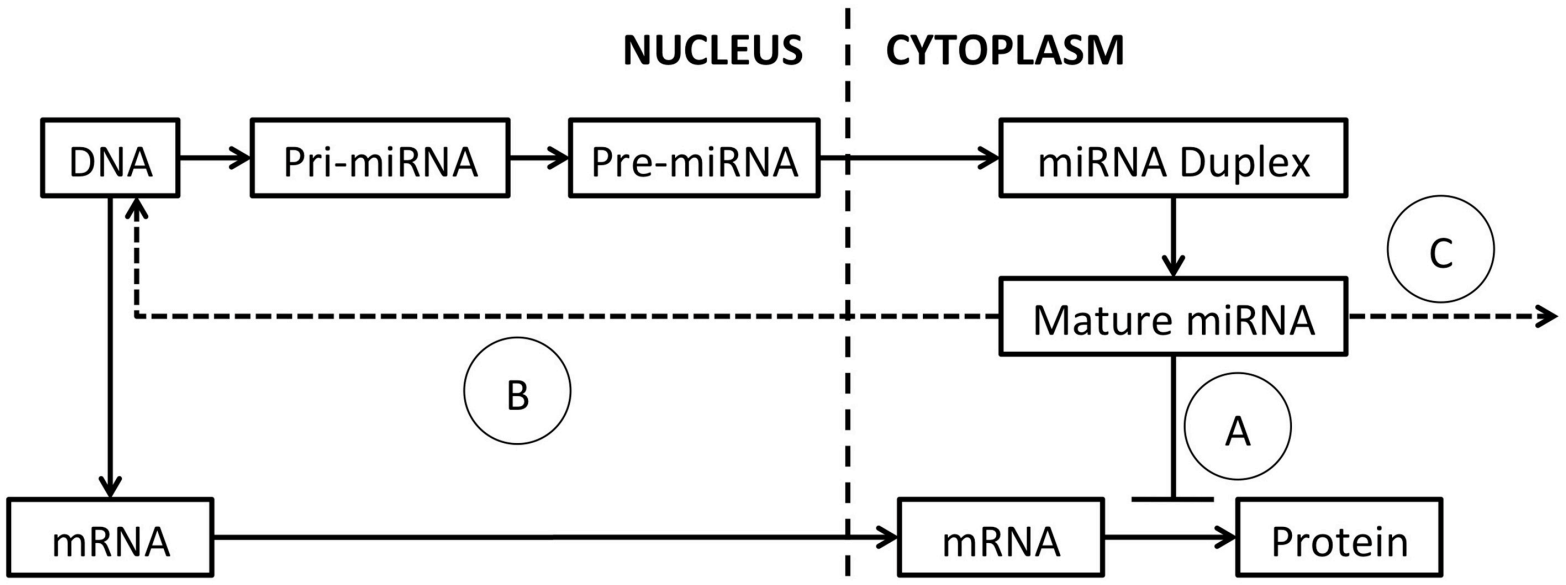
**MiRNA processing and function**. The primary miRNA transcript (pri-miRNA) is transcribed from DNA and excised by Drosha, to produce the pre-miRNA. The pre-miRNA is exported to the cytoplasm by exportin-5 and spliced by Dicer to generate a miRNA duplex. The duplex is unwound by helicases and a mature single-stranded miRNA is assembled into the RISC complex. (A) MiRNA typically modulate target mRNA translation by complementary binding, within the 3′ UTR, 5′ UTR or coding region. New evidence suggests that miRNAs also function by (B) binding of DNA promoters, acting as RNA decoys or through direct binding of target mRNAs. (C) Mature miRNAs can also be released in exosomes and act on distant cells.

The mechanisms regulating miRNA expression, processing and function have been extensively reviewed (Ha and Kim, 2014) and a schematic of miRNA processing and function is presented in Figure 1. MiRNAs are commonly encoded either within the introns of protein-coding genes or as independent genes, and transcribed by RNA polymerase II (Lee et al., 2004). After transcription, mature miRNAs are generated through a multi-step process (Lee et al., 2002). First, the primary transcript (pri-miRNA) is processed by the nuclear RNase III Drosha-DGCR8 (DiGeorge syndrome critical region gene 8) complex, into a ~65 nucleotide hairpin precursor miRNA (pre-miRNA; Denli et al., 2004). Pre-miRNAs are exported from the nucleus into the cytoplasm by exportin 5 and cleaved by the RNase III enzyme Dicer into a double-stranded miRNA-miRNA^*^ duplex approximately 22 nucleotides (nt) in length (Hutvágner et al., 2001; Ketting et al., 2001). This miRNA duplex is unwound by helicases and a mature miRNA is incorporated into the RNA-induced silencing complex (RISC; Bartel, 2004). The RISC complex binds target mRNA sequences through partially complementary binding with the incorporated miRNA (Bartel, 2004; Weinmann et al., 2009).

MiRNAs typically modulate target mRNA levels by binding to the 3′ untranslated region (UTR) of mRNA transcripts (Hammond et al., 2001). This occurs through complementary binding of the highly specific seed sequence at the 5′ end of a miRNA to the target mRNA (Mallory et al., 2004). Sequence complementarity between the rest of the miRNA and the target mRNA is often quite low, allowing individual miRNAs to target multiple mRNA sequences and making the prediction of miRNA targets difficult (Lewis et al., 2003; Mallory et al., 2004; Lim et al., 2005; Liu, 2008). MiRNA:mRNA interactions repress protein translation and/or reduce target mRNA stability, resulting in decreased protein translation from the target mRNA.

New studies are adding increased complexity to our understanding of how miRNAs function. In addition to binding target sites within the 3′-UTR of mRNA transcripts, miRNA can also bind within the 5′-UTR (Lytle et al., 2007), or coding regions (Forman et al., 2008). Further, a subset of miRNAs increase target expression through a number of mechanisms (Vasudevan et al., 2007), including binding DNA promoters (Place et al., 2008), acting as RNA decoys (Eiring et al., 2010) or through binding of target mRNAs (Ørom et al., 2008). MiRNAs can also be released from expressing cells and detected in exosomes in the circulation allowing communication between neighboring and distant cell populations (Mitchell et al., 2008; Liu et al., 2010a; Redis et al., 2012). As miRNAs are single-stranded RNA molecules, they may also activate immune cells following binding to Toll-like receptors (TLRs) either as part of their function, or as an unintended consequence following administration (Fabbri et al., 2012; Lehmann et al., 2012).

## MiRNA expression in respiratory diseases

Key roles for miRNAs in normal lung development and respiratory diseases have been extensively reviewed (Plank et al., 2013; Rupani et al., 2013; Booton and Lindsay, 2014). These include extensive reviews of specific respiratory diseases including asthma (Tay et al., 2014), chronic obstructive pulmonary disease (COPD) (De Smet et al., 2015), cystic fibrosis (CF) (Sonneville et al., 2015), and idiopathic pulmonary fibrosis (IPF) (Pandit et al., 2011), as well as lung cancer (Lin et al., 2010; Rusek et al., 2015). For the purpose of this mini-review, we will briefly summarize recent findings on miRNA expression and function in each of these diseases (Table 1).

**Table 1:** **MicroRNA profiling studies and individual microRNAs identified in respiratory diseases**.

**Respiratory diseases**	**miRNA expression profiling**	**Individual miRNAs**
Asthma	Patient samples: Bronchial epithelium (Jardim et al., 2012; Solberg et al., 2012) BALF exosomes (Levänen et al., 2013) Peripheral blood (Panganiban et al., 2012; Yamamoto et al., 2012) Blood: child population (Liu et al., 2012) Airway T cells (Simpson et al., 2014) Mouse models of allergic airways disease: Three models (Garbacki et al., 2011) Deep sequencing lung (Polikepahad et al., 2010)	Functional roles: let-7 (Polikepahad et al., 2010) miR-9 (Li et al., 2015) miR-19a (Simpson et al., 2014) miR-21 (Lu et al., 2009, 2011) miR-106a (Sharma et al., 2009, 2012) miR-126 (Mattes et al., 2009; Collison et al., 2011a) miR-145 (Collison et al., 2011b) miR-155 (Malmhäll et al., 2014; Okoye et al., 2014) miR-221 (Qin et al., 2012)
COPD	Smoking-induced changes: Isolated lung macrophages (Graff et al., 2012; Gross et al., 2014) Lung tissues (Izzotti et al., 2009a,b) Bronchial epithelium (Schembri et al., 2009) Lung tissue: Smokers (COPD vs. healthy) (Ezzie et al., 2012) Related to emphysema severity (Savarimuthu Francis et al., 2014) Regions related to emphysema (Christenson et al., 2013) Biomarkers: Sputum (Van Pottelberge et al., 2011) Exhaled breath condensates (Pinkerton et al., 2013)	Biomarkers: Serum miR-7 (Akbas et al., 2012) Serum miR-21, -181a (Xie et al., 2014) *In vitro:* miR-146a (Sato et al., 2010) miR-199a-5p (Mizuno et al., 2012; Chatila et al., 2014; Hassan et al., 2014) Functional roles: miR-135b (Halappanavar et al., 2013) miR-144, -101 (Hassan et al., 2012a)
CF	Endobronchial brushings (Oglesby et al., 2010)	Targeting CFTR: miR-101, -494 (Megiorni et al., 2011) miR-138 (Ramachandran et al., 2012) miR-145, -223, -494 (Oglesby et al., 2013) miR-509-3p, -494 (Ramachandran et al., 2013) Inflammation: miR-17 (Oglesby et al., 2015) miR-126 (Oglesby et al., 2010) miR-145 (Megiorni et al., 2013) miR-155 (Bhattacharyya et al., 2011)
IPF	Patient lungs (Pandit et al., 2010) Serum (Li et al., 2014)	Functional roles: Let-7 (Pandit et al., 2010) miR-21 (Liu et al., 2010b; Li et al., 2013) miR-26a (Liang et al., 2014) miR-29 (Cushing et al., 2011) miR-155 (Pottier et al., 2009) miR-200 (Yang et al., 2012) miR-326 (Das et al., 2014) miR-486 (Ji et al., 2015)

*COPD, chronic obstructive pulmonary disease, CF, cystic fibrosis, IPF, idiopathic pulmonary fibrosis*.

### Asthma

Many studies have performed miRNA profiling on samples from patients with asthma. Profiling of bronchial epithelial cells identified 60–200 differentially expressed miRNAs (Jardim et al., 2012; Solberg et al., 2012), and isolated exosomes from bronchial alveolar lavage fluids exhibited changes in 24 miRNAs (Levänen et al., 2013). Profiling of peripheral blood samples revealed alterations in miRNA expression patterns, with miR-192 notably decreased (Yamamoto et al., 2012), and alterations in miR-124, -26a, let-7a, and let-7d (Panganiban et al., 2012). Profiling of circulating lymphocytes also identified upregulation of miR-221 and miR-485-3p in asthmatic children compared to healthy controls (as well as in an ovalbumin (OVA)-induced mouse model of asthma; Liu et al., 2012). Profiling of airway T cells also revealed increased levels of miR-19a in patients with asthma, with functional roles in T_H_2 cytokine production (Simpson et al., 2014).

A growing number of studies have also demonstrated alterations and functional roles for miRNAs in mouse models of allergic airways disease. A profiling study using three murine models of allergic inflammation identified a number of altered miRNAs, including miR-29b, -29c, -146b, -223, -483, -574-5p, -672, and -690 (Garbacki et al., 2011). Deep sequencing of mouse lungs revealed dynamic changes in miRNA expression following OVA-induced allergic airway inflammation and demonstrated high levels of let-7 family members in OVA-challenged lungs (Polikepahad et al., 2010). Inhibition of let-7 family members reduced the allergic phenotype and IL-13 expression (Polikepahad et al., 2010). MiR-21 expression is increased in several models of experimental asthma (Lu et al., 2009) and *mir-21* gene deletion reduced T_H_2 responses and decreased eosinophilia in an ovalbumin (OVA)-induced asthma model (Lu et al., 2011). Inhibition of miR-106a also reduced features of disease including AHR, inflammation and fibrosis (Sharma et al., 2009, 2012). *Mir-155*-deficient mice also have decreased asthma disease severity (Malmhäll et al., 2014), which was attributed to altered T cell function (Okoye et al., 2014). Inhibition of miR-221 in a mouse model of asthma also suppresses airway inflammation (Qin et al., 2012).

Our group demonstrated that antagomir-mediated inhibition of miR-126 significantly reduced airway hyperreactivity, eosinophil recruitment, mucus hypersecretion, and T_H_2 cell activation (Mattes et al., 2009). However, while inhibition of miR-126 reduced eosinophil infiltration in chronic asthma models, it failed to inhibit inflammation and airway remodeling (Collison et al., 2011a). Expression of miR-145, miR-21, and let-7b were increased after house dust-mite (HDM) exposure and inhibition of miR-145 (but not miR-21 or let-7b) suppressed airways inflammation (Collison et al., 2011b). Recently, we also demonstrated that inhibition of miR-9 restored steroid sensitivity and dampened airways inflammation in an otherwise steroid-resistant airways disease model (Li et al., 2015).

Respiratory bacterial and viral infections are also associated with asthma onset and disease exacerbations (reviewed in Falsey and Walsh, 2000; Hansbro et al., 2004; Friedlander and Busse, 2005; Piedimonte, 2013; Starkey et al., 2013; Choroszy-Król et al., 2014; Leigh and Proud, 2015). For example, *Haemophilus influenzae* bacterial infection is linked to both exacerbations and disease severity in patients with asthma (Wood et al., 2010). Importantly, key roles for miRNA functions are recognized in responses to respiratory infections [reviewed for virus (Globinska et al., 2014) and bacterial infections (Staedel and Darfeuille, 2013)]. For example, inhibition of miR-328 promoted clearance of *H. influenzae*, even in the context of steroid-induced immunosuppression (Tay et al., 2015). Thus, modulation of miRNA function may impact asthma severity by altering the course of respiratory infections and miRNAs may also serve as therapeutic targets for the management of infection-induced asthma exacerbation.

### Chronic obstructive pulmonary disease

A growing number of studies have also assessed miRNA expression in COPD patients. As smoking is a major contributing factor for COPD development, many studies have also assessed impacts of cigarette smoke exposure alone. However, these studies have not yet assessed functional impacts in COPD.

Cigarette smoke reduced global miRNA expression, in human alveolar macrophages isolated from smokers compared to non-smokers (Graff et al., 2012; Gross et al., 2014) and lung tissue from smoke-exposed mice or rats (Izzotti et al., 2009a,b). In isolated bronchial airway epithelium from smokers, 28 miRNAs were differentially expressed, including miR-218 (Schembri et al., 2009). Profiling of miRNAs from different lung regions in smokers with COPD revealed subsets of miRNAs that correlated with regional emphysema severity (Christenson et al., 2013). In a comparison of lung tissue from smokers with COPD, to smokers without COPD, miRNA profiling revealed increased levels of 57 miRNAs, with 13 decreased (Ezzie et al., 2012). Further, altered miRNA expression in lung tissue was detectable between COPD patients based on emphysema severity (Savarimuthu Francis et al., 2014).

MiRNA expression has been proposed as an accessible biomarker of COPD disease. The ratio of serum miR-21 to -181a was predictive for COPD in asymptomatic heavy smokers (Xie et al., 2014). Decreased serum levels of miR-20a, -28-3p, -34c-5p, and -100, and increased miR-7 were also detected in COPD patients (Akbas et al., 2012). Similarly, altered miRNA expression in sputum (Van Pottelberge et al., 2011) and exhaled breath condensates (Pinkerton et al., 2013) have been detected in patients with COPD.

A number of individual miRNAs have also been assessed in samples isolated from COPD patients. MiR-146a induction following *ex vivo* cytokine activation is reduced in fibroblasts from COPD patients that smoke, compared to fibroblasts from smokers without COPD (Sato et al., 2010). MiR-199a-5p was decreased in peripheral blood monocytes from symptomatic COPD patients (Hassan et al., 2014), with decreases also observed in circulating regulatory T cells (Chatila et al., 2014). However, levels of miR-199-5p were increased in total lung tissues (Mizuno et al., 2012). In mouse models, miR-135b was increased in lungs from smoke-exposed mice, which required interleukin 1 (IL-1)-R1 expression and acted as a negative regulator of IL-1 activation and signaling (Halappanavar et al., 2013). In lungs from smoke-exposed mice and COPD patients, miR-144 and -101 were also both increased, and suppressed expression of their target CF transmembrane conductance regulator (CFTR) (Hassan et al., 2012a).

### Cystic fibrosis

Cystic fibrosis is caused by mutations in the CFTR gene. Despite this simple cause, significant heterogeneity exists between CF patients, suggesting potential roles for epigenetic regulation, including miRNA alterations (Cutting, 2010).

Most work on miRNA function in CF has assessed direct impacts on CFTR expression. Increased miR-101 and -494 repressed CFTR expression in cell lines *in vitro* (Megiorni et al., 2011). Further, miR-138 overexpression increased levels of CFTR (via suppression of its target SIN3 transcription regulator family member A, in CF airway epithelial cells (Ramachandran et al., 2012). MiR-145, -223, and -494 were increased in CF bronchial brushings, and directly regulated CFTR expression *in vitro* (Oglesby et al., 2013). MiR-509-3p and -494 were also increased in primary cultured airway epithelia from CF patients, vs. non-CF controls, and cooperatively repressed CFTR expression (Ramachandran et al., 2013).

Several studies have also assessed miRNA-mediated regulation of inflammation in CF patients. MiR-17 was decreased in CF bronchial brushings, resulting in increased expression of its pro-inflammatory target, IL-8 (Oglesby et al., 2015). MiR-126 was downregulated in bronchial epithelial cells from CF patients, with significant increases in target of myb protein 1, which modulates inflammatory responses (Oglesby et al., 2010). MiR-155 was increased in mutant CFTR epithelial cell lines, contributing to increased pro-inflammatory IL-8 release (Bhattacharyya et al., 2011). Finally, elevated miR-145 levels negatively correlated with its target SMAD family member 3 in nasal epithelial cells from CF patients, potentially regulating downstream transforming growth factor, beta 1 (TGF-β1) inflammatory pathways (Megiorni et al., 2013).

In addition to direct effects on CFTR and inflammation, bacterial infections worsen CF disease symptoms and contribute to long-term airway remodeling. Thus, impacts of miRNA function on anti-bacterial immunity may also regulate disease progression and shape disease management strategies.

### Idiopathic pulmonary fibrosis

IPF is a chronic fibrosing interstitial lung disease with unknown causes. Initial profiling of IPF lungs revealed that 10% of lung-expressed miRNAs were altered, compared to normal controls (Pandit et al., 2010). Profiling of circulating serum miRNAs in patients with IPF revealed altered expression of miR-21, -155, and -101-3p associated with clinical features of disease (Li et al., 2014). MiR-21 expression was increased in the serum of IPF patients, and levels correlated with decreases in lung function (Li et al., 2013). Let-7 was also significantly downregulated in IPF lungs and may contribute to fibrosis through regulation of high mobility group A2 (Pandit et al., 2010).

Little is known about miRNA function in IPF and importantly, much of the functional data has been inferred from chemically-induced mouse models of lung fibrosis. MiR-29 was reduced in mouse models, as seen in IPF lung samples (Pandit et al., 2010), and knockdown in human fetal lung fibroblasts increased fibrosis-associated gene expression (Cushing et al., 2011). MiR-21 expression was increased in myofibroblasts from IPF lungs and inhibition in mouse fibrosis models dampened disease severity (Liu et al., 2010b). MiR-155 was increased in mouse fibrosis models (Pottier et al., 2009), and may modulate fibrosis through regulation of angiotensin II type I receptor, which is increased in IPF lungs (Königshoff et al., 2007). MiR-200 family members were reduced in mouse models of lung fibrosis and restoration of miR-200c inhibited fibrosis (Yang et al., 2012). MiR-26a was decreased in fibrosis models and inhibition caused pulmonary fibrosis, while overexpression repressed fibrotic disease (Liang et al., 2014). MiR-326 was also decreased in mouse fibrosis models and human IPF lung samples, and administration of miR-326 mimics inhibited TGF-β expression and dampened fibrosis (Das et al., 2014). MiR-486-5p was decreased in lung tissues of patients with IPF and overexpression of miR-486-5p limited fibrosis in mouse models (Ji et al., 2015).

### Lung cancer

The impacts of miRNA dysregulation on cancer initiation and progression have been extensively studied and reviewed (Hayes et al., 2014; Lin and Gregory, 2015). A summary of the proposed roles for miRNAs in cancer is beyond the scope of this mini-review and we direct readers to reviews that have summarized miRNA functions in lung cancers specifically (Lin et al., 2010; Rusek et al., 2015).

## Targeting MiRNA function in therapy

The studies described have revealed changes in miRNA expression in respiratory diseases and key functions across a range of disease processes. Interestingly, miRNA-targeted interventions modified disease pathogenesis in pre-clinical disease models, for a small subset of the miRNAs identified in each disease.

Based on similar findings in other fields, many biopharmaceutical companies are now targeting miRNAs using novel therapeutics. The first drug to inhibit a specific miRNA (miR-122) entered Phase II clinical trials in 2010 for the treatment of hepatitis C virus infection (Janssen et al., 2013). The first drug to increase a specific miRNA (miR-34 mimic; MRX34) entered Phase I clinical trials in 2013, for the treatment of advanced hepatocellular carcinoma (Bouchie, 2013). However, no clinical trials have yet assessed the impacts of miRNA-targeted strategies in respiratory diseases.

## Pharmacological activation of MiRNA function

One approach to alter miRNA function *in vivo* is through administration of double-stranded synthetic miRNA oligonucleotides, termed mimics, which have been used extensively in cancer studies (Henry et al., 2011). These double-stranded molecules are processed by the endogenous miRNA processing machinery, integrated into the RISC complex and dampen target mRNA translation. A main limitation of this approach is the effective delivery of mimic molecules into target cells.

One strategy to improve cell targeting is the coupling of mimics to antibody-coated nanoparticles, as used in neuroblastoma (Liu et al., 2011). Another approach (used in the current MRX34 clinical trial), is encapsulation of mimic molecules in liposome-based delivery system to facilitate target cell uptake (Bouchie, 2013).

An alternative approach that stably increases miRNA levels is delivery of miRNA expression vectors (Stegmeier et al., 2005). This approach can yield stable, long-term miRNA expression and provides the potential to target vectors to specific cell types or enforce cell-type specific miRNA expression using specific promoters.

## Pharmacological inhibition of MiRNA function

To inhibit target miRNAs, oligonucleotide inhibitors with complementary sequences to the miRNA of interest can be used (anti-miRs). These molecules are often comprised of locked nucleic acid backbones with specific modifications to increase stability *in vivo* (Lennox and Behlke, 2011). One specific category of inhibitor, termed antagomirs, is further modified with a 2′-*O*-methyl linkage and phosphorothioate modification to improve binding efficiency and prevent nuclease degradation (Krützfeldt et al., 2005). Anti-miR treatments interfere with target miRNA function, increasing translation of miRNA-targeted mRNAs. Antagomir-mediated miRNA-silencing *in vivo* is dose-dependent and can last for several weeks after a single administration (Krützfeldt et al., 2005).

Another strategy to inhibit miRNA function is the use of “miRNA sponges.” MiRNA sponges are transgenes that encode RNA molecules comprised of multiple tandem miRNA target sites (Ebert et al., 2007). The repeated target sequences compete with endogenous mRNA targets for RISC binding, reducing the impact of a specific miRNA on its intended target (Ebert et al., 2007). This approach has been used extensively *in vitro* and in preclinical animal models. However, this approach also requires delivery of a transgene-encoding vector to achieve stable inhibition (Gentner et al., 2009).

## Considerations for MiRNA-targeted interventions in respiratory diseases

There are a number of considerations and issues to consider when modulating miRNA expression in general, and more specifically in lung disease (reviewed in Hassan et al., 2012b).

Individual miRNAs modulate expression of multiple mRNA targets and interfering with single miRNAs can have broad effects on multiple cellular pathways. For this reason, therapies targeting individual miRNAs can have broader impacts than traditional single-molecule/single-target approaches. Further, compared to short-interfering RNA approaches, miRNA targeting may have broader effects on more mRNAs, but more moderate effects on each individual mRNA (Baker, 2010). This can be beneficial, as individual miRNAs often regulate multiple genes in a related gene network. However, the alteration of multiple downstream targets also increases the likelihood of undesired side effects, particularly if systemic drug delivery is used.

The simplest approaches to modulate miRNA levels are through administration of “naked” nucleic acids (either mimic or anti-miR). This approach provides the advantage of being able to tailor dosing and withdraw treatment if complications arise. In respiratory diseases, direct tissue administration by aerosol provides targeted delivery to lung tissue, with minimal dissemination into the circulation and systemic tissues. This is likely to limit side effects, compared to systemic delivery. However, this also means that these therapies will only be effective if altering miRNA function specifically within the lung is sufficient to dampen disease processes. Even with direct administration into the lung, efficient delivery to target cell populations remains difficult, as seen in siRNA-based therapies (Lam et al., 2012). For example, we observe cell type-specific differences in antagomir distribution following intranasal administration *in vivo*, with efficient uptake in myeloid cell populations and poor targeting of lymphocytes, which was also replicated *in vitro* (Plank et al., 2015).

More complex treatment strategies requiring stable expression and viral delivery systems (e.g., sustained miRNA delivery or miRNA sponges) add additional complications relating to the expression vector itself. Viral delivery platforms have been associated with direct toxicity, increased risks associated with genomic integration events and the inability to discontinue treatment if problems do arise (Ibrahim et al., 2011). A further drawback, specific to miRNA modulation using viral delivery systems, is toxicity induced by the overwhelming of the exportin-5 pathway following enforced miRNA expression (Castanotto et al., 2007).

## Conclusions

Increasing numbers of publications have identified differences in miRNA expression and functional roles for miRNAs across a range of respiratory diseases. MiRNAs have been proposed as biomarkers of disease and potential novel therapeutic targets for treatment. In respiratory diseases, direct administration of miRNA-targeting drugs into the lungs may allow tissue-specific targeting, while limiting side effects resulting from systemic delivery. Continued mechanistic studies are required to optimize drug delivery systems and demonstrate acceptable efficacy and safety for translation into the clinic. While miRNA-targeting approaches hold promise, no studies have yet assessed impacts in human respiratory diseases.

## Author contributions

SM, MP, HT, AC, and PF wrote and edited the manuscript.

### Conflict of interest statement

The authors declare that the research was conducted in the absence of any commercial or financial relationships that could be construed as a potential conflict of interest.
